# Adaptive population structure shifts in invasive parasitic mites, *Varroa destructor*

**DOI:** 10.1002/ece3.7272

**Published:** 2021-05-01

**Authors:** Arrigo Moro, Tjeerd Blacquière, Bjørn Dahle, Vincent Dietemann, Yves Le Conte, Barbara Locke, Peter Neumann, Alexis Beaurepaire

**Affiliations:** ^1^ Vetsuisse Faculty Institute of Bee Health University of Bern Bern Switzerland; ^2^ Swiss Bee Research Center Agroscope Bern Switzerland; ^3^ Wageningen University & Research Wageningen Netherlands; ^4^ Norwegian Beekeepers Association Kløfta Norway; ^5^ Faculty of Environmental Sciences and Natural Resource Management Norwegian University of Life Sciences Ås Norway; ^6^ Department of Ecology and Evolution University of Lausanne Lausanne Switzerland; ^7^ UR 406 Abeilles et Environnement INRAE Avignon France; ^8^ Department of Ecology Swedish University of Agricultural Sciences Uppsala Sweden

**Keywords:** coevolution, genetic diversity, host–parasite interactions, invasive species, population genetics, population structure

## Abstract

Comparative studies of genetic diversity and population structure can shed light on the ecological and evolutionary factors governing host–parasite interactions. Even though invasive parasites are considered of major biological importance, little is known about their adaptative potential when infesting the new hosts. Here, the genetic diversification of *Varroa destructor*, a novel parasite of *Apis mellifera* originating from Asia, was investigated using population genetics to determine how the genetic structure of the parasite changed in distinct European populations of its new host. To do so, mites infesting two categories of hosts in four European regions were compared: (a) adapted hosts surviving through means of natural selection, thereby expected to impose strong selective pressure on the mites, and (b) treated host populations, surviving mite infestations because acaricides are applied, therefore characterized by a relaxed selection imposed by the host on the mites. Significant genetic divergence was found across regions, partially reflecting the invasion pattern of *V. destructor* throughout Europe and indicating local adaptation of the mite to the host populations. Additionally, varying degrees of genotypic changes were found between mites from adapted and treated colonies. Altogether, these results indicate that *V. destructor* managed to overcome the genetic bottlenecks following its introduction in Europe and that host‐mediated selection fostered changes in the genetic structure of this mite at diverse geographic scales. These findings highlight the potential of parasites to adapt to their local host populations and confirm that adaptations developed within coevolutionary dynamics are a major determinant of population genetic changes.

## Graphical abstract

We investigated the genetic structure of the honey bee ectoparasite *Varroa destructor* across sympatric adapted and nonadapted populations in four regions of Europe. Our results reveal that host‐mediated selection fostered changes in the genetic structure of this mite at diverse geographic scales. These findings highlight the potential of parasites to adapt to their local host populations and confirm that adaptations developed within coevolutionary dynamics are a major determinant of population genetic changes.

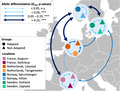

## 1 INTRODUCTION

Coevolution is a process of reciprocal evolutionary changes between interacting species (Ehrlich & Raven, 1964; Thompson, 2005). Adaptations developed within host–parasite interactions strongly shape the genetics of the coevolving species (Anderson & May, 1982; Thompson, 2005; Woolhouse et al., 2002). Notably, the rate of genetic changes is expected to be particularly swift in parasite populations compared to their hosts because of their shorter generation time (Paterson et al., 2010; Schmid‐Hempel, 2011). Yet, the selection of specific parasite adaptations is not uniform for parasites infesting distinct populations of hosts, because different selective forces may act on these populations (Gandon & Zandt, 1998). These forces, composed of diverse elements of environmental factors or unique host population adaptations, generally result in the genetic diversification of parasite populations at varying geographic scales, thereby generating geographic mosaics of coevolution (Thompson, 2005).

Although adaptations of parasites to their host traits have been studied extensively in silico (Gandon & Michalakis, 2002) and in vitro (Brockhurst & Koskella, 2013), evidences of mosaics of coevolution in invasive parasites infesting novel hosts remain scarce. The relatively recent coevolving system between the Western honey bee, *Apis mellifera*, and the obligate ectoparasitic mite, *Varroa destructor,* represents an ideal opportunity to investigate the impact of host adaptations on parasite evolution in real time (Dietemann et al., 2012; Oldroyd, 1999). Originally, *V. destructor* infested colonies of Eastern honey bees, *Apis cerana*, in Asia (Oldroyd, 1999; Rath, 1999). However, the introduction of *A. mellifera* colonies in the native range of the parasite resulted in the mite shifting host in the middle of the 20th century, and in its spread to almost all locations where beekeeping with the Western honey bee is practiced (Traynor et al., 2020). The spread of these parasites was particularly swift in Europe. Having been first detected in eastern regions of the continent in the beginning of the 1970s, it had dispersed throughout most of the continent just two decades later (Rosenkranz et al., 2010).

Out of the many *V. destructor* haplotypes found in *A. cerana* colonies (Navajas, 2010), only two are known to have switched to *A. mellifera* and to have emigrated from Asia (Anderson & Trueman, 2000). In addition to this original bottleneck, and coupled with the incestuous mating behavior of the mite (Rosenkranz et al. 2010), the host shift and subsequent spread of the pest has resulted in a highly homogenous genetic population structure within the invasive populations of *V. destructor* (Solignac et al., 2005). Despite this low genetic diversity, *V. destructor* has flourished as an invasive parasite, with devastating consequences for its new host (Neumann & Carreck, 2010; Potts, Biesmeijer, et al., 2010) and the quasi‐eradication of wild honey bee populations in Europe (Jaffé et al., 2010; Moritz et al., 2007). In *A. mellifera* colonies, mite population growth is exponential (Martin, 1998) and, due to its ability to vector lethal honey bee viruses (Beaurepaire et al., 2020; Traynor et al., 2020), survival of susceptible colonies is dependent on annual mite control treatments implemented by beekeepers (Boecking & Genersch, 2008; Le Conte et al., 2010).

These regular mite treatments may remove the selective pressure imposed by the parasite (May & Anderson, 1990; Schmid‐Hempel, 2011), thereby limiting the full potential of host adaptations (Fries & Camazine, 2001; Neumann & Blacquière, 2017) as well as the selection pressure of the host on *V. destructor*. Only occasionally were populations of European Western honey bees in France, Sweden, The Netherlands, and Norway left untreated and had the opportunity to adapt by means of natural selection (Fries et al., 2006; Kruitwagen et al., 2017; Le Conte et al., 2007; Locke, 2016; Oddie et al., 2017; Panziera et al., 2017). These adapted small honey bee populations, in France, The Netherlands, Norway, and Sweden, are considered resistant as they are surviving without the need for acaricide treatments by expressing a wide variety of traits that enable them to interfere with *V. destructor* population growth (Locke, 2016; Mondet et al. 2020).

Given that populations of *V. destructor* were genetically homogenous upon their introduction in Europe (Solignac et al., 2005), the adaptation potential of this invasive parasite has received little attention (Eliash & Mikheyev, 2020). Yet, the reduced diversity and common origin of the recently introduced mites represents a perfect starting point to study how adaptation to novel host populations and different environments can induce genetic diversification in the parasite. More specifically, when coevolving with adapted *A. mellifera* colonies, mites would be expected to swiftly develop counter‐adaptations against the resistance traits of the hosts in order to survive. Such adaptations should result in changes in the levels of genetic diversity and population structure of the mites (Schulte et al., 2010, 2013). For instance, the length of brood production during the season, which is a trait known to enable honey bee survival to *V. destructor* (Locke, 2016), may influence the yearly number of mite reproductive cycles and the level of recombination of inbred mite lineages (Beaurepaire et al., 2017). In contrast, mites infesting susceptible colonies that require regular treatments may face different selective forces. In this case, treatments may relax the selective pressure of mite infestation on the hosts but are instead a strong selective pressure on the parasite population, which could also lead to the development of resistance toward treatments (González‐Cabrera et al., 2018; Martin, 2004; Milani, 1999). Consequently, the genetic structure of *V. destructor* populations infesting honey bee colonies that are adapted to the mite versus those that are susceptible and require mite control for survival may follow different evolutionary paths and result in a geographic mosaic of coevolution (Thompson, 2005). To investigate this hypothesis, we performed an analysis of the genetic diversity and population structure of mites infesting five adapted and five treated honey bee populations located in four European regions. Our results show significant genetic diversification across *V. destructor* populations in the studied regions and host populations, thereby suggesting that mites have overcome the initial bottleneck of their introduction and are adapting to their local host populations as predicted by the geographic mosaic of coevolution theory (Thompson, 2005).

## 2 MATERIALS AND METHODS

### 2.1 Sampling

In this study, adapted honey bee populations are defined as groups of colonies that have survived *V. destructor* infestations without the need for treatments for more than ten years, and do not require treatments to survive for extended periods. In contrast, the susceptible host populations are defined as treated since they require frequent treatments and/or management practice in order to survive mite infestations. Such treated populations generally show significantly lower expression levels of mite resistance traits compared to adapted ones (Mondet et al. 2020). In 2017, adult female *V. destructor* mites were sampled from 32 treated and 28 sympatric adapted *A. mellifera* colonies, in various locations across four different regions: France, Netherlands, Sweden, and Norway (Tables 1 and 2). Mite samples were initially divided into 11 groups (Table 1), reflecting the apiary location and type of colony they were infesting (i.e., adapted or treated).

**TABLE 1:** Sampling overview

Region	Location (coordinates)	Group	Treatment	*N* colonies	*N* mites
France	Avignon (43°54′56.3″N, 4°52′39.4″E)	Adapted (1)	None	6	171
	Avignon (43°54′56.1″N, 4°52′37.7″E)	Treated	Amitraz	6	168
	Solérieux (44°20′40″N, 4°49′33.2″E)	Treated	Amitraz	8	90
Netherlands	Tiengemeten (51°43′56″N, 4°20′54″E)	Adapted (2)	None	6	195
	Lelystad (52°32′09″N, 5°32′21″E)	Adapted (2)	None	6	143
	Lelystad (52°32′8.42″N, 5°32′20.02″E)	Treated	Oxalic Acid	6	96
Norway	Sørumtangen (60°03′12.6″N, 11°05′26.8″E)	Adapted (5)	None	5	175
	Hilton (60°04′12.1″N, 11°07′13.3″E)	Treated	Oxalic Acid	4	107
Sweden	Gotland (57°4′7.3″N, 18°12′27.0″E)	Adapted (3–4)	None	5	53
	Gotland (57°22′27.0″N 18°40′24.3″E)	Treated (4)	Oxalic Acid	4	38
	Uppsala (59°49′4.9″N, 17°39′22.9″E)	Treated (4)	Oxalic Acid	4	74
Total				60	1,310

Note

Region, location, and origin of experimental colonies, acaricide treatment occurrence and type, number of sampled hives, and number of mites genotyped are shown. Some of the mite groups listed are numbered to provide references to previous studies (1. Le Conte et al., 2007, 2. Panziera et al., 2017, 3. Locke, 2016, 4. Beaurepaire et al., 2019, and 5. Oddie et al., 2017).

**TABLE 2:** Sampled locations and distances between adapted and treated apiaries in each country

Region	Location 1	Location 2	Spatial distance (km)	Genetic distance (*D*_est_)
France	Avignon (Ad, 1)	Avignon (Tr)	0.01	0.029***
France	Solérieux (Tr)	Avignon (Tr)	45	0.009***
Netherlands	Tiengemeten (Ad, 2)	Lelystad (Tr)	100	0.103***
Netherlands	Lelystad (Ad, 2)	Lelystad (Tr)	0.01	0.081***
Norway	Sørumtangen (Ad, 5)	Hilton (Tr)	2.5	0.009***
Sweden	Gotland (Ad, 3–4)	Uppsala (Tr, 4)	325	0.021***
Sweden	Gotland (Ad, 3–4)	Gotland (Tr, 4)	10	0.028***
Sweden	Uppsala (Tr, 4)	Gotland (Tr, 4)	325	0.041***

Note

A significant but low positive correlation was found between spatial and genetic distance (Mantel test, *R* ^2^ = 0.1393; *p* =.001). Codes between brackets indicate the mite groups (Ad: adapted, Tr: treated), and numbers provide references to previous studies (1. Le Conte et al., 2007, 2. Panziera et al., 2017, 3. Locke, 2016, 4. Beaurepaire et al., 2019, and 5. Oddie et al., 2017).

*** Stands for highly significant p‐values (*p* < 0.001).

The treatments of the treated colonies differed across the studied regions (Table 1). The adapted and treated colonies were located in the same apiary at two locations (Avignon, France; Lelystad, Netherlands). At all other locations, the distance between treated and surviving colonies ranged from 2.5 to 325 km (Table 2). The mites (*N* = 1,310) were collected on adult workers using standard methods (i.e., powdered sugar, Dietemann et al., 2013) during the summer of 2016 and 2017 and were immediately transferred into 95% EtOH and stored at −20°C until DNA extraction.

### 2.2 DNA extraction and genotyping

Established protocols were followed to isolate total mite DNA (Beaurepaire et al., 2017). In brief, mites were washed twice in ddH_2_0 to remove the EtOH and then individually distributed in 96‐well plates filled with 100 µl of Chelex™ solution. Individual mites were then crushed with sterile pipette tips, 5 µl of 10 mg/ml Proteinase K was added, and their DNA was extracted following Walsh et al. (2013).

Initially, 20 microsatellites (Beaurepaire et al., 2017; Cornman et al., 2010; Evans, 2000) were tested on 12 individual mites from each location (*N* = 132) to assess the genetic diversity and population structure of the *V. destructor* samples. PCRs were conducted as detailed in Beaurepaire et al. (2019). Seven of the tested markers were polymorphic over all regions and were thus chosen for genotyping all samples (*N* = 1,310, Table 3). Twenty‐four mites were genotyped for each colony whenever the infestation level allowed to. PCR products were sent to Genoscreen (Lille, France) to run on a 3730XL sequencer (Applied Biosystems®, Carlsbad, CA). All mites were genotyped using the Peak Scanner TM software v 1.0 (Applied Biosystems®, Carlsbad, CA).

**TABLE 3:** General information on the microsatellite primers used for the analysis

Name	Reference	*T*_A_	Size	*N*_A_	*H*_O_
VD307	Cornman et al. (2010)	60	162	2	0.059
Vj292	Evans (2000)	60	233	4	0.005
Vj294		58	170	4	0.027
Vj295		58	150	4	0.002
Vdes01	Beaurepaire et al. (2017)	60	400	4	0.065
Vdes02		60	296	2	0.041
Vdes03		60	303	2	0.043

Note

The annealing temperature (*T*_A_) and average fragment size (Size, bp) of the primers as well as the number of alleles (*N*_A_) and average heterozygosity (*H*_O_) scored during the analysis are listed.

### 2.3 Microsatellite DNA analyses

In total, 1,310 mites were individually genotyped at seven polymorphic loci (Table 1). Notably, the data from Sweden (165 mites) have already been published in Beaurepaire et al. (2019) and are used here to compare the amplitude of genetic changes across distinct mite groups. To verify the independence of the markers used, all locus pairs were tested for linkage disequilibrium using the software Fstat V 2.9.3 (Goudet, 1995).

To assess genetic differences between *V. destructor* infesting adapted versus susceptible *A. mellifera* populations in the different locations, the mites were initially grouped depending on apiary location and the type of host colonies they infested (i.e., adapted or treated; Table 1). To confirm this a priori sample clustering, the levels of genetic variance across sampling regions and between host categories (i.e., treated versus adapted) were tested using an analysis of molecular variance (AMOVA, Excoffier & Smouse, 1992) performed in R v. 3.5.1 (R Core Team, 2018) with the *poppr* package (Kamvar et al., 2014).

Genetic diversity estimates, including number of alleles (*N*_A_) and observed heterozygosity (*H*_O_), were compared using Fstat v. 2.9.3 (Goudet, 1995). These results were statistically compared using Kruskal–Wallis tests using R v. 3.5.1 (R Core Team, 2018). In order to understand whether the lowest sample size used in this study (i.e., Gotland treated mites, *N* = 38) was suitable to accurately represent the genetic diversity of the mite groups, rarefaction analyses were conducted using the ADZE software (Szpiech et al., 2008), using the allelic richness averages obtained across all markers in each mite group.

To test the allelic divergence of *V. destructor* across regions and between honey bee groups within locations, estimates of *D*_est_ (Jost, 2008) and pairwise tests of population differentiation were obtained for each pair of location (i.e., grouping individuals from the different host populations in each location) and within location for each possible adapted versus treated comparison using the software GenAlex v. 6.5 (Peakall & Smouse, 2012). The statistical significance of the pairwise population divergence indexes was obtained using Fstat v. 2.9.3 (Goudet, 1995) after 55,000 permutations, as this software allows to correct for multiple comparisons. Additionally, the level of genetic differentiation (*D*_est_) between mite groups treated with different acaricides was compared using a Student *t* test. Finally, to investigate the relationships between the genetic distance obtained with *D*_est_ and the spatial distance separating the mite groups (see Table 2), a Mantel test of correlation (Mantel, 1967) was performed with GenAlex v. 6.5. For mite groups that were present on the same apiary (i.e., Avignon, France, and Lelystad, The Netherlands), a distance of 10 meters was considered.

Finally, the diversity and the prevalence of mite genotypes infesting the different honey bee populations were compared. To do so, the distribution of multilocus genotypes (MLGs) in each mite group was computed using the R package *poppr* (Kamvar et al., 2014). Comparison of the diversity of MLGs across adapted and treated populations was done by estimating the 95% confidence intervals of Shannon (Shannon & Weaver, 1949) and Simpson (Simpson, 1949) diversity indexes using 1,000 bootstraps. In parallel, the differences in the distribution of the most common MLGs between the adapted and treated host populations within each region were tested. To do so, only the mite genotypes with a frequency of at least 5% in the considered locations were compared using Fisher exact test conducted using contingency tables in R. As tests were conducted pairwise between the populations of any given region, the p‐values obtained were corrected for multiple comparisons using Holm's method (Holm, 1979). In this analysis, all individuals with missing data were excluded from the dataset, resulting in a total of 863 individuals.

## 3 RESULTS

No significant linkage disequilibrium between pairs of markers was detected after correction for multiple comparisons (all *p*‐values > .05). The AMOVA confirmed our a priori grouping, indicating that sampling regions and host groups (i.e., treated versus adapted) were significantly (*p* <.01) structuring *V. destructor* populations (i.e., responsible for 41.9% and 7.6% of the total genetic variance observed, respectively; Table 4).

**TABLE 4:** AMOVA results

Variation	Sigma	%	*p*‐Value
Between Region	1.185	41.89	**
Between Group within Region	0.187	6.62	**
Within groups	1.456	51.48	**

Note

Variations between hierarchical grouping levels are reported. Levels of significance are indicated with stars (**p‐value = .01). Only samples with less than 5% missing values (*N* = 863) were considered in this analysis.

**FIGURE 1:**
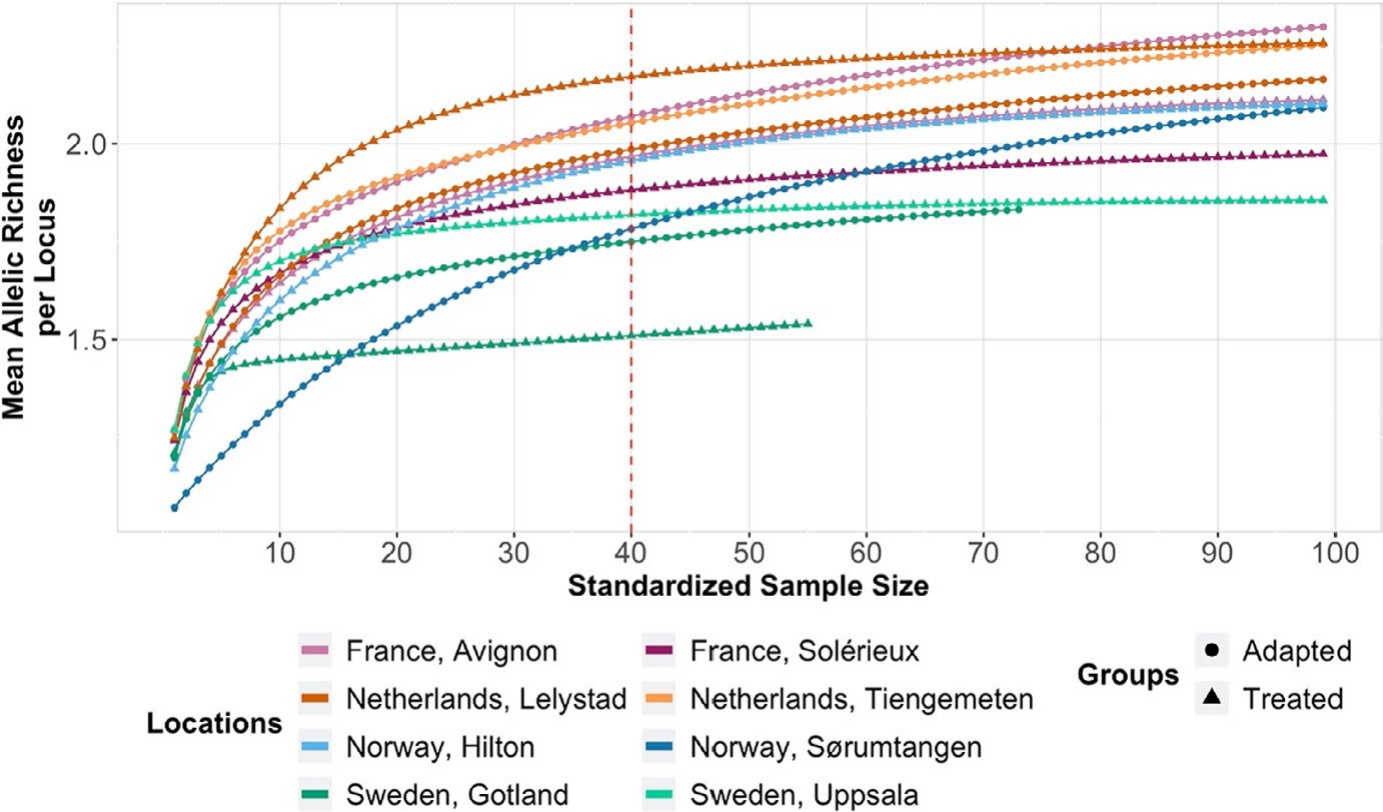
Rarefaction analysis. Mean allelic richness as a function of the standardized sample size calculated with ADZE (Szpiech et al., 2008) for all the mite groups analyzed. The red dash line indicates the threshold, identified by the rarefaction analysis, below which the sample size is considered insufficient to capture the genetic diversity of the groups

**FIGURE 2:**
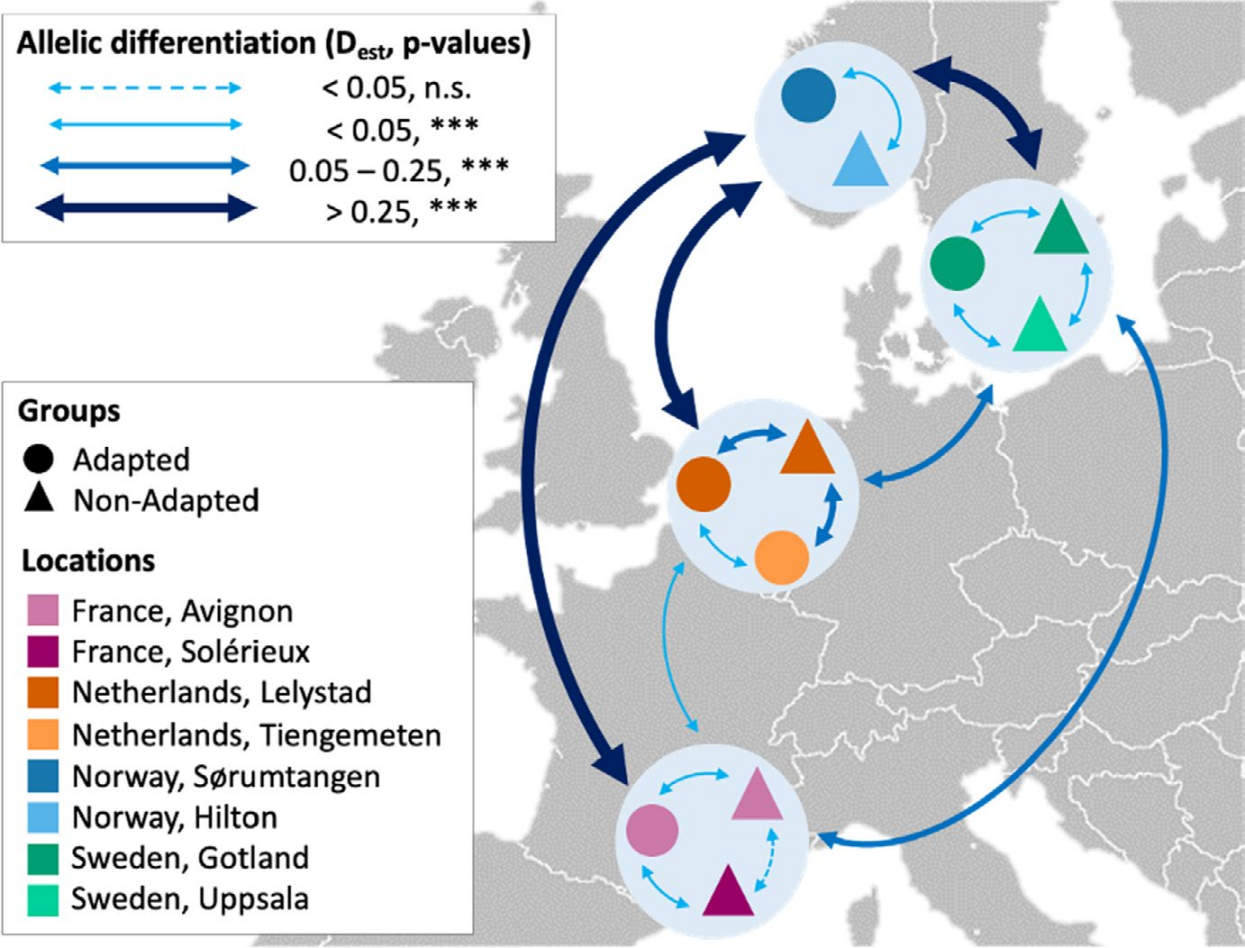
*Varroa destructor* population structure across locations and groups of colonies. Results of the tests of allelic divergence (*D*_est_) between mites infesting colonies of *A. mellifera* at the four locations and between treated and adapted colonies within each location. The thickness of the arrows linking populations shows the level of allelic divergence between them, from low (*D*_est_ < 0.05) to high (*D*_est_ > 0.25), while the dashed and solid lines represent statistical nonsignificance and significance, respectively (*** indicates *p* <.001). For every region, significant population structure divergences were found when adapted and treated mites were compared (*p* <.001). Notably, Norwegian mites were genetically highly isolated from all the other mite groups (*D*_est_ = 0.32–0.38)

Variable, but low levels of observed heterozygosity (*H*_o_ = 0.002–0.065) and number of alleles (NA = 2–4) were found across the seven markers over all samples (Table 3). For all mite groups, the rarefaction analyses showed that allelic richness only increased marginally after ~ 40 mites were analyzed (Figure 1). The comparison of allelic divergence of *V. destructor* populations in Europe revealed diverging patterns across the sampled regions. This analysis revealed very low allelic divergence between France and the Netherlands (*D*_est_ = 0.01, *p* <.001), while mites from Sweden were more markedly differentiated from mites of these two regions (*D*_est_ = 0.12, *p* <.001). Surprisingly, the genetic divergence levels between samples from these three locations and mites from Norway were thrice as high (*D*_est_ = 0.32–0.38, *p* <.001; Figure 2).

**FIGURE 3:**
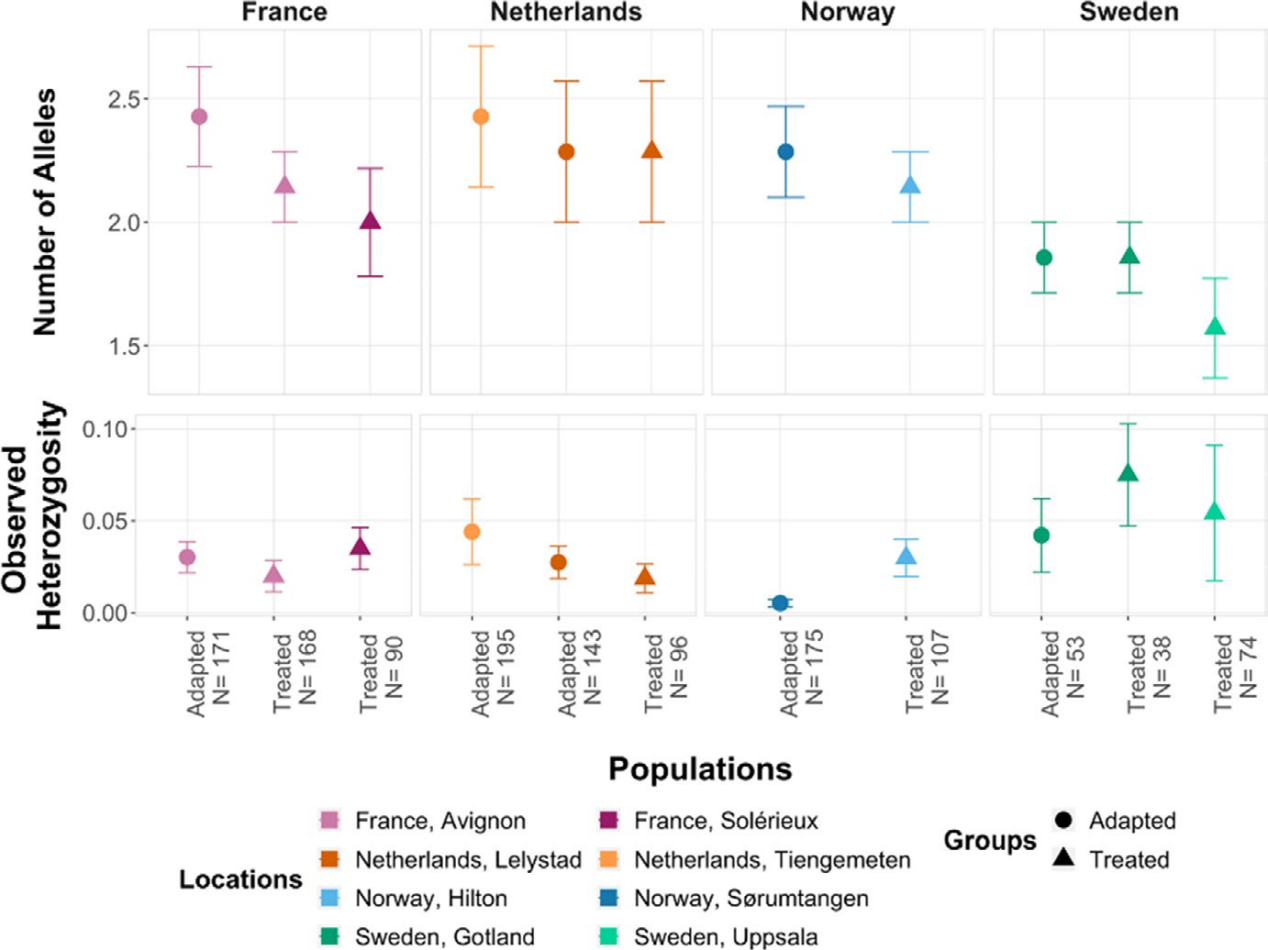
Estimates of genetic diversity. Mean (± standard error) number of alleles and observed heterozygosity for each group of mites. Mites were grouped by location (countries are distinguished by color and locations within countries by color shades) and by the type of colony they infested (designated by symbols). No significant difference was found when mite groups were compared across regions and between populations of the same region (Kruskal–Wallis test, *p* > .05)

**FIGURE 4:**
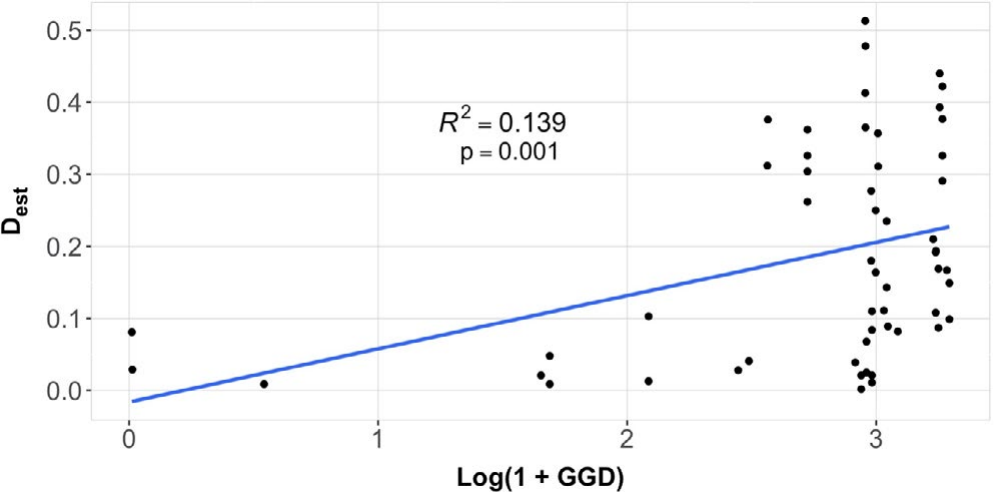
Correlation between geographic distance (GGD) and genetic distance (*D*_est_). Results of the Mantel analysis of the spatial and genetic distance separating the mite groups. A weak but significant correlation (*p* =.001) was found between the distances

The comparison of genetic diversity across mite groups infesting the different host populations revealed that the mean number of alleles and the observed heterozygosity levels did not differ significantly between these groups (Kruskal–Wallis tests, *p* >.05; Figure 3). However, the levels of genetic differentiation between mites sampled from adapted and treated populations within each region resulted all significant, with diverse *D*_est_ levels (Figure 2). Notably, the degree of genetic differentiation weakly but significantly correlated with the distance separating the groups (Mantel test, *R* ^2^ = 0.14, *p* =.001; Figure 4). However, looking more closely at the pairwise comparisons between mite infesting different host groups revealed that mite genetic differentiation is not clearly determined by geographic distance. For instance, the level of divergence between mites in the two treated French populations was very low (*D*_est_ = 0.01) and nonsignificant (Table 5) despite the distance separating these two groups (45 km). In contrast, higher and significant differences (*D*_est_ = 0.029–0.048, *p* <.001) were found when these two groups were compared with the mites infesting the French adapted population, despite the fact that one of the treated host group was in the same apiary as the adapted honey bees. Also, the lowest level of divergence in the Netherlands occurred between the two adapted groups (*D*_est_ = 0.013, *p* <.001), in spite of the ~100 km separating them. In parallel, the pairwise allelic comparisons revealed that mites from the adapted host population in France and from Tiengemeten in the Netherlands were not significantly genetically different (*D*_est_ = 0.002, *p* =.056, Table 5), while mites infesting susceptible populations in these two regions were (*D*_est_ = 0.07–0.08; *p* <.001, Table 5). Moreover, the average level of population divergence found between mites treated with Amitraz and mites groups treated with oxalic acid (average *D*_est_ = 0.21, ±0.21 *SD*) did not differ significantly from that obtained when mite groups treated with oxalic acid were compared with each other (average *D*_est_ = 0.27, ±0.14 *SD*, *t* test, *p* =.63).

**TABLE 5:** Pairwise population divergence indexes (*D*_est_)

France	Ad Avignon	Tr Avignon
Tr Avignon	0.029***	
Tr Solérieux	0.048***	0.009 n.s.

Netherlands	Ad Tiengemeten	Ad Lelystad
Ad Lelystad	0.013***	
Tr Lelystad	0.103***	0.081***

Sweden	Ad Gotland	Tr Gotland
Tr Gotland	0.021***	
Tr Uppsala	0.028***	0.041***

Norway	Tr Hilton
Ad Sørumtangen	0.009***

Regions	France	Netherlands	Sweden
Netherlands	0.007***		
Sweden	0.124***	0.124***	
Norway	0.366***	0.383***	0.324***

Adapted	France Avignon	Netherlands Tiengemeten	Netherlands Lelystad	Sweden Gotland
Netherlands Tiengemeten	0.002 n.s.			
Netherlands Lelystad	0.021***	0.013***		
Sweden Gotland	0.087***	0.089***	0.164***	
Norway Sørumtangen	0.326***	0.357***	0.413***	0.362***

Treated	France Avignon	France Solérieux	Netherlands Lelystad	Sweden Uppsala	Sweden Gotland
France Solérieux	0.009 n.s.				
Netherlands Lelystad	0.084***	0.068***			
Sweden Gotland	0.192***	0.21***	0.277***		
Sweden Uppsala	0.149***	0.167***	0.235***	0.041***	
Norway Hilton	0.377***	0.393***	0.478***	0.262***	0.312***

Note

Results of pairwise population differentiation indices across countries when pooling all mites of a given region (overall), comparing treated colonies (Tr), and comparing adapted colonies (Ad). Numbers indicate *D*_est_ value, and stars and “n.s.” indicate *p*‐value of the test (***: *p* <.001, n.s.: nonsignificant). Values are color‐coded according to the degree of *D*_est_ (red = high, yellow = moderate, and green = low).

The analysis of multilocus genotypes (MLGs) showed 139 distinct mite genotypes over all groups (Table 6). A total of 36 genotypes were shared between the two host population (i.e., adapted and treated) and represented the majority of the samples (74.5%). Additionally, 48 genotypes, representing 11.1% of the total number of mites included in the MLG analysis, were only found in adapted colonies across all locations, while 55 (14.4%) were found only in treated populations. Several private MLGs (i.e., MLGs found only in one group) were detected in every group. Their numbers (*N* = 3–13) varied between groups, but only represented a minor proportion of the sampled mites (0.5%–4.1%, Table 6).

**TABLE 6:** Comparison of the mite multilocus genotypes

Region	Group	Number of MLGs and proportion of mites		Number of private MLGs and proportion of mites	
France	Avignon (Ad)	77 (30.2%)	32 (9.73%)	23 (6.62%)	9 (1.27%)
	Avignon (Tr)		23 (11.94%)		8 (1.16%)
	Solérieux (Tr)		22 (8.57%)		3 (0.46%)
Netherlands	Tiengemeten (Ad)	92 (29.08%)	41 (10.78%)	37 (8.57%)	13 (1.97%)
	Lelystad (Ad)		27 (11.12%)		8 (1.04%)
	Lelystad (Tr)		24 (7.18%)		13 (4.06%)
Norway	(Ad)	32 (27%)	12 (15.64%)	18 (3.94%)	4 (0.58%)
	(Tr)		20 (11.36%)		12 (1.85%)
Sweden	Gotland (Ad)	56 (13.67%)	15 (4.17%)	21 (3.48%)	10 (1.51%)
	Gotland (Tr)		28 (6.84%)		7 (1.16%)
	Uppsala (Tr)		13 (2.67%)		3 (0.58%)
Overall	(Ad)	127 (51.45%)		48 (11.12%)	
	(Tr)	130 (48.55)		55 (14.37%)	

Note

The number and proportion of mite multilocus genotypes (MLGs) across the locations and groups of honey bee colonies are shown. The number of private MLGs exclusive to each group of mites is also reported. The codes between brackets indicate the mite groups (Ad: adapted, Tr: treated), and numbers provide references to previous studies (1. Le Conte et al., 2007, 2. Panziera et al., 2017, 3. Locke, 2016, 4. Beaurepaire et al., 2019, and 5. Oddie et al., 2017), while the percentages represent the proportion of individuals out of the 863 mites included in this analysis.

**FIGURE 5:**
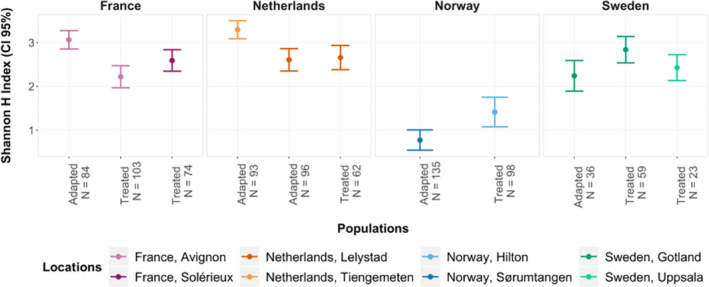
Comparison of MLG diversity (Shannon Index). Graph representing 95% confidence interval of the Shannon index, illustrating the diversity of multilocus genotypes (MLGs) across mite groups. The diversity analysis was conducted after excluding all mites with missing data. The sample size after this exclusion is reported for every group. Overall, contrasting patterns of MLG diversity could be observed between adapted and treated populations across the different locations. Significantly higher levels of MLG diversity were found in mites infesting adapted host populations in Avignon (France) and Tiengemeten (the Netherlands) compared to the mite infesting treated hosts from their respective region. Notably, for Norwegian mites, a significantly lower level of MLG diversity was found in the adapted group compared to treated one, whereas for Swedish mites, no significant difference was found

**FIGURE 6:**
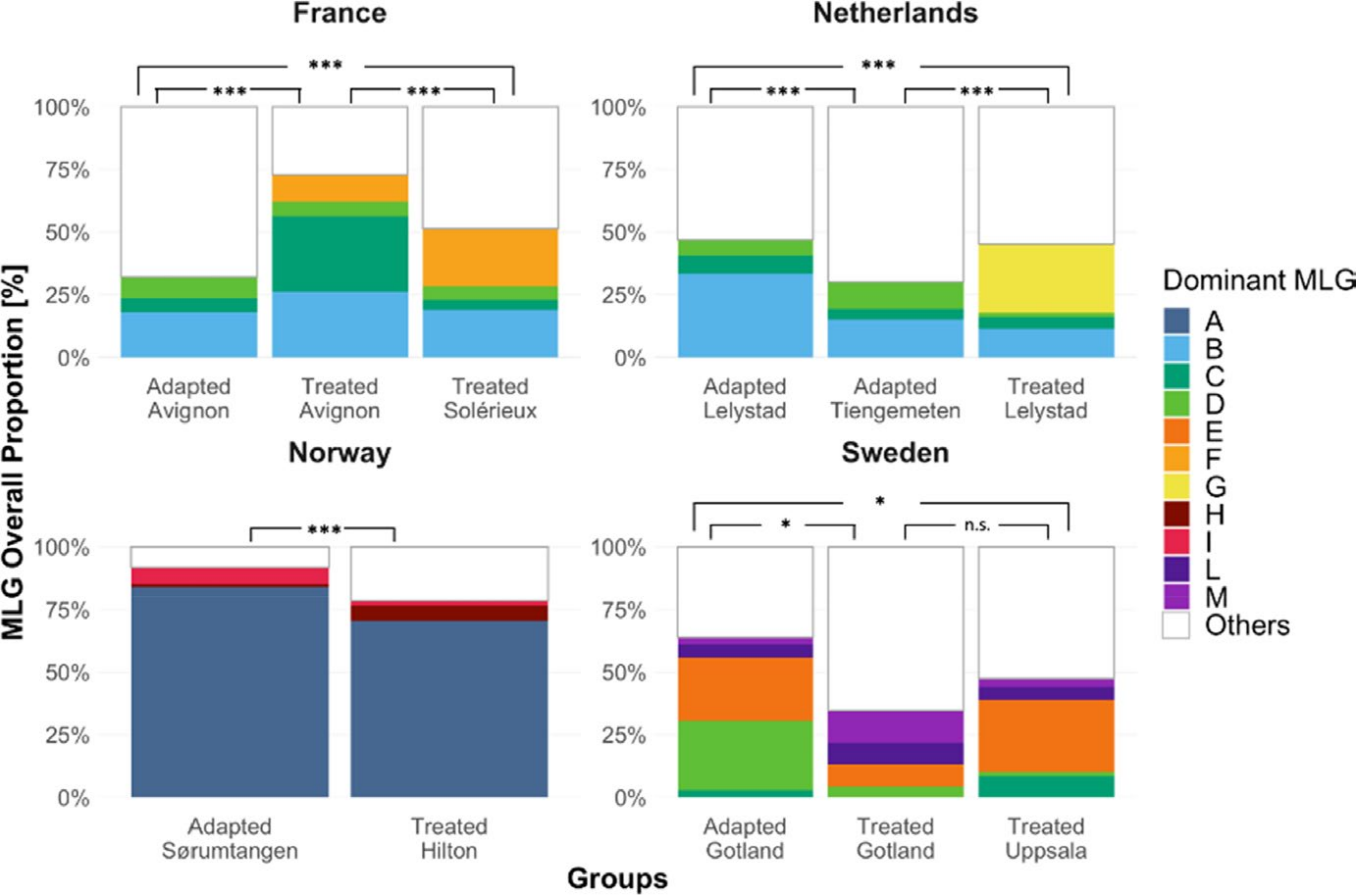
Distribution of the dominant mite genotypes. Frequency of the dominant mite multilocus genotypes (MLGs) at each location for adapted and treated *A. mellifera* colonies. Dominant MLGs are defined by a frequency >5% at the given location and are represented by different colors. The less frequent MLGs were pooled and designated as “Others.” Mites with missing data were excluded from this analysis. The stars indicate the significance level of *p*‐values obtained using Fisher exact test calculated with contingency table coupled with Holm's correction for multiple comparison (*p < .05, ***p < .001), while n.s. indicates nonsignificance. The distribution of mite genotypes varied significantly when adapted and treated mite groups were compared in every region (Holm's adjusted *p* < .05)

Overall, contrasting patterns of MLG diversity could be observed when comparing mites from adapted and treated populations across the different locations (Figure 5). Notably, in France, significantly higher levels of the Shannon index (H) were found in mites infesting the adapted host population (95% CI = 2.9–3.3) compared to the two mite groups sampled from the treated populations of the same region (95% CI = 2.0–2.5 and 2.4–2.8). The same pattern was found in one adapted population of the Netherlands (95% CI = 3.1–3.5) when compared to the adapted (95% CI = 2.4 – 2. 8) and treated populations (95% CI = 2.4–2.9) from the same region. In addition, the distribution of the dominant MLGs varied significantly between adapted and treated colonies in each location (Fisher exact tests corrected for multiple testing, *p* <.05, Figure 6).

## 4 DISCUSSION

Our results suggest that the genetic structure of European *V. destructor* is shaped by their interactions with their local host populations. These findings reveal that sympatric and allopatric mite populations are experiencing significant levels of genetic divergence that are probably caused by both unique host population traits and environmental differences across locations.

A weak and significant interaction (*R* ^2^ = 0.14, *p* =.001) was found between the genetic and geographic distance separating the mite groups, indicating that geographic isolation can explain a small part of the genetic differences found between distant mite populations. Additionally, the pattern of genetic differentiation documented here appears to be further explained by the invasion history of the parasite in Europe. Historical reports of this invasion showed that *V. destructor* arrived in Europe through two routes. First, the mite is believed to have been introduced in 1971–72 in Eastern European countries and to slowly spread to the Northern regions of the continent, including Sweden (Griffiths & Bowman, 1981; Rosenkranz et al., 2010). Secondly, the parasite was introduced in Germany in 1977, a source from which mites from France and Netherlands derived (Nixon, 1983; Potts, Roberts, et al., 2010; Ruttner & Ritter, 1980). After its introduction in Europe, the trading of honey bee colonies between regions became restricted and subjected to strict regulations (European Commission, 1992), so to limit the spread of the parasite across countries. Our results match these previous reports of introduction as the low genetic differentiation between mites infesting French and Dutch honey bees suggests that these populations derived from a common origin, while more elevated levels of differentiation between these two locations and Sweden suggest a distinct introduction. However, the level of differentiation between mites from these three regions compared to Norwegian mites was much higher, pointing to a third introduction event. This hypothesis matches reports on the arrival of *V. destructor* in Norway, which was first found in the spring of 1993 in Oslo, while the natural spread of the mite was still confined to the southernmost part of Sweden. *V. destructor* was in fact found for the first time in Sweden on the island of Gotland in 1987 (Fries, 1987) and later in Malmö, on the southern Swedish mainland, in the spring of 1991 (Fries, 1991). Notably, the first identification of *V. destructor* infesting colonies along the Norwegian–Swedish border was reported in 1995, two years after the arrival of the mite in Norway, and was considered to be caused by a natural spread of the parasites from Norwegian apiaries. To date, the precise origin of the mites infesting Norwegian honey bee colonies remains to be identified. Nevertheless, this is the first report of a previously unknown route of introduction of *V. destructor* into Europe. Notably, the marked differentiation of these mites from all the other groups analyzed here may suggest that the Norwegian *V. destructor* originate from a different source population.

Over all the *V. destructor* populations sampled, relatively low numbers of alleles and heterozygosity levels were detected. However, the number of alleles detected appears to be higher than initially reported by other authors investigating the genetic diversity of *V. destructor* infesting several European honey bee populations with microsatellites (Solignac et al., 2005). When genotyping 92 mites from Avignon with 13 polymorphic markers over a decade ago, these authors obtained a total of 1.3 alleles per marker. In the present study, taking place some 15 years later, 171 and 168 mites were sampled in two honey bee groups from Avignon, yielding more alleles per markers (i.e., an average of 2.4 and 2.1 alleles per marker). This temporal increase does not seem to be caused by the different sample sizes used between the two studies, as the rarefaction analysis showed that a sample size of 40 mites is enough to accurately quantify the diversity of invasive populations of *V. destructor*. Additionally, the analyses performed in the current study further suggest that *V. destructor* populations have diversified since their introduction in Europe, as shown by the high differences between and within locations from the same countries. When comparing the distribution of mite genotypes, a relatively high number of MLGs (*N* = 139 MLGs out of 1,310 mites genotyped) were found across the honey bee populations, including many rare MLGs private to specific regions and populations. Notably, the distribution of MLGs significantly differed across the populations studied, and strong and significant differences were also found when performing pairwise allelic differentiation analyses between regions. Altogether, these results suggest that the mites are adapting to their local host populations, despite the relatively recent genetic bottlenecks caused by both the host shift and introduction of the mite in Europe (Solignac et al. 2005).

In addition to the diversification of *V. destructor* across Europe, the comparison between mites infesting sympatric adapted and treated host colonies revealed intriguing patterns of genetic structure. Although the specific mechanisms of horizontal large‐scale transmission of the parasite currently remain unknown, the mite can easily spread within and between apiaries of a given region (Frey et al., 2011; Fries & Camazine, 2001). In molecular terms, this high transmission may prevent genetic isolation of mites infesting honey bee colonies within and across apiaries (Beaurepaire et al., 2015, 2017; Dynes et al., 2017). Despite this, we here observed significantly different allelic patterns and MLG distribution between *V. destructor* samples infesting adapted and treated honey bee colonies in all the four regions studied. These differences may be explained by numerous factors. First, neutral processes such as genetic drift may cause isolated mite populations to diverge (Freeland et al., 2011). However, the differences reported here do not seem to result entirely from this factor as the patterns of genetic differentiation have been found to be consistently dependent on mite–host association in every location (Figures 2 and 5). Although a weak isolation by distance at the continental scale was found, the patterns of genotypic differences between *V. destructor* infesting adapted and treated populations did not vary according to the distance separating the groups in a given region. For example, differences remained significant even in locations where the two host populations were located at the very same apiary (i.e., Lelystad, the Netherlands, and Avignon, France).

Second, acaricide treatments of the susceptible colonies could have affected the population structure of the mites. The application of pesticides is known to lead to the development of resistance in pest populations (Georghiou, 1972), *V. destructor* being no exception (Martin, 2004; Milani, 1999; Spreafico et al., 2001). Although we here did not test directly for the presence of acaricide resistance, the variability of microsatellites markers in response to pesticide‐driven population genetic changes can provide indirect evidence for changes in genotypic diversity and structure caused by pesticides (de Meeûs et al., 2007; Osakabe et al., 2009; Pascual‐Ruiz et al., 2014). Notably, the use of acaricides should reduce population sizes and diversity levels. As a consequence, an increase of the level of genetic divergence between treated and nontreated populations may also occur (Osakabe et al., 2009; Uesugi et al., 2009). Here, the number of alleles and the levels of heterozygosity did not differ significantly between mites from adapted and treated colonies, but the diversity of mite MLGs and/or the amplitude of genetic divergence varied between these groups across populations. Notably, three of the four treated populations examined in this study (Netherlands, Sweden, and Norway) have been regularly treated with oxalic acid to control *V. destructor* infestations. This organic acaricide has high and consistent efficacy (Gregorc & Planinc, 2001) and works through contact by killing mites by means of high acidity (Nanetti, 1999). Given this very general mode of action, oxalic acid is not expected to select for any particular lineage of mites, as it has been demonstrated in a previous study, where even after a repeated and prolonged exposure to this compound, mites remained susceptible (Maggi et al., 2017). In contrast, the synthetic acaricide (i.e., Amitraz) used to treat the susceptible colonies in Avignon and Solérieux can foster the development of resistance in *V. destructor* (Kamler et al., 2016) and could have caused some of the genetic differences between mites from adapted and treated host populations in France. However, the number of alleles and level of heterozygosity of mites infesting treated hosts in France was as low as in the other mite groups. Moreover, the average level of population divergence obtained in mites treated with Amitraz versus oxalic acid and in populations treated with oxalic acid only did not differ significantly. Altogether, these findings suggest that the acaricide treatments of the susceptible colonies did not greatly affect the genetic diversity and population structure of *V. destructor*.

Another factor that may explain the results documented here lies in the natural adaptations of honey bee colonies to *V. destructor*. Most strikingly, the pairwise comparisons revealed that mites from adapted colonies from France and one location of the Netherlands (Tiengemeten) were not significantly genetically different, while the mite infesting susceptible colonies in these two regions were. This may suggest parallel evolution similar to their honey bee hosts (Locke et al., 2012; Oddie et al., 2018). On the other hand, the patterns of change in the diversity of MLGs between mites infesting the adapted and treated host populations were not consistent across regions. For instance, in the populations located in France and the Netherlands, a higher diversity of MLGs was observed in the adapted colonies compared to local treated ones. In contrast, the level of MLG diversity was higher in the treated colonies from Norway and was not significantly different between the three groups located in Sweden. Possibly, in some surviving populations, host‐mediated selection may promote mite genotypes expressing a decreased level of reproduction (i.e., selection for lower parasite virulence; Seeley, 2007), while in others, the hosts may select for mite genotypes having specific chemical mimicry abilities (Kather et al., 2015; Le Conte et al., 2015). These results suggest that different selective forces may be acting on the various mite groups, thereby representing hot spots and cold spots of evolution as postulated by the geographic mosaic of coevolution theory (Thompson, 2005). Although the particular host traits shaping the population structure of *V. destructor* remain to be discovered, these results confirm previous findings documenting significant temporal changes of population structure between parasites sampled in adapted and susceptible honey bee colonies over nine years (Beaurepaire et al., 2019). Altogether, these results provide empirical evidence that honey bee selective pressure influences *V. destructor* population structure, as previously observed in other systems (birds‐fleas: Alves et al., 2019; donkeys‐helminths: Decaestecker et al., 2007; kangaroos‐worms: Koskella & Lively, 2009).

In conclusion, the data presented here show that the genetic structure of *V. destructor* populations differs across European regions and across sympatric groups of varroa mite‐adapted and treated hosts, resulting in a geographic mosaic of coevolution between the ectoparasitic mites and their hosts in different populations throughout Europe. These findings shed new light into the interactions between *V. destructor* and *A. mellifera*, and highlight the so far underestimated role of the mite adaptations in this system (Eliash & Mikheyev, 2020). Half a century after its introduction into Europe, the ongoing genetic diversification of *V. destructor* in Europe illustrates well the evolutionary potential of parasites and represents a prominent example of ongoing coevolution between hosts and parasites.

## CONFLICT OF INTEREST

The authors declare no conflict of interest.

## AUTHOR CONTRIBUTIONS

**Arrigo Moro:** Data curation (lead); Formal analysis (lead); Visualization (lead); Writing‐original draft (lead); Writing‐review & editing (lead). **Tjeerd Blacquière:** Conceptualization (equal); Data curation (supporting); Methodology (equal); Validation (supporting); Writing‐original draft (supporting); Writing‐review & editing (supporting). **Bjorn Dahle:** Conceptualization (equal); Data curation (supporting); Methodology (equal); Validation (supporting); Writing‐original draft (supporting); Writing‐review & editing (supporting). **Vincent Dietemann:** Conceptualization (supporting); Investigation (supporting); Methodology (supporting); Writing‐original draft (supporting); Writing‐review & editing (supporting). **Yves Le Conte:** Conceptualization (equal); Investigation (supporting); Methodology (equal); Validation (supporting); Writing‐original draft (supporting); Writing‐review & editing (supporting). **Locke Barbara :** Conceptualization (equal); Data curation (supporting); Methodology (equal); Validation (supporting); Writing‐original draft (supporting); Writing‐review & editing (supporting). **Peter Neumann:** Conceptualization (equal); Funding acquisition (lead); Methodology (equal); Project administration (equal); Resources (lead); Writing‐original draft (supporting); Writing‐review & editing (supporting). **Alexis Beaurepaire:** Conceptualization (lead); Data curation (equal); Formal analysis (supporting); Methodology (lead); Project administration (supporting); Supervision (lead); Validation (lead); Writing‐original draft (lead); Writing‐review & editing (lead).

## Supporting information

Supplementary Material

## Data Availability

The dataset used in this study can be found here: https://datadryad.org/stash/share/e_WmWloADcFQjut8RpxoVZ2StvUVdfL30hts2bd7PFA.
